# Supplementary material for: Spatiotemporal Patterns of Tuberculosis in Hunan Province, China
Source: Int J Environ Res Public Health. 2021 Jun 24;18(13):6778. doi: 10.3390/ijerph18136778 (PMC8297355; doi:10.3390/ijerph18136778)
Supplement: Supplementary file 1 [file ijerph-18-06778-s001.zip › ijerph-1102250-supplementary.pdf]

Supporting information

Table S1: The non-spatial, spatial, and both the spatial and non-spatial models constructed using WinGUGS to estimate the posterior parameter.

Model I: Non-spatial model	Model II: Spatial model	Model III: both spatial and non-spatial
$Yi \sim$ Poisson (μ_i);<br>Log (μ_i) = Log $Ei + \theta i$;<br>$\theta i = \alpha + \beta_{1i} X_{1i} + \ldots \beta_{ni} X_{ni} + \gamma t + U_i$;<br>$U_i \sim$ dnormal (0.0, *tau*);<br>Beta (β_{1i}) ~ dnorm (0.0,1.0E-6);<br>Alpha (α) ~ dnorm (0.0,1.0E-4);<br>*tau* ~ dgamma (0.001, 0.001);	$Yi \sim$ Poisson (μ_i);<br>Log (μ_i) = Log $Ei + \theta i$;<br>$\theta i = \alpha + \beta_{1i} X_{1i} + \ldots \beta_{ni} X_{ni} + \gamma t + V_i$;<br>$V_i \sim$ *car. Normal* (*adjacencyi, weighti, numberi, tau.v*);<br>Beta (β_{1i}) ~ dnorm(0.0,1.0E-6);<br>Alpha (α) ~ dflat() ;<br>*tau.v* ~ dgamma(0.001, 0.001);	$Yi \sim$ Poisson (μ_i);<br>Log (μ_i) = Log $Ei + \theta i$<br>$\theta i = \alpha + \beta_{1i} X_{1i} + \ldots \beta_{ni} X_{ni} + \gamma t + U_i + V_i$;<br>$U_i \sim$ dnormal (0.0, *tau*);<br>$V_i \sim$ *car. Normal* (*adjacencyi, weighti, numberi, tau.v);*<br>Beta (β_{1i}) ~ dnorm(0.0,1.0E-6);<br>Alpha (α) ~ dflat() ;<br>*tau.u* ~ dgamma(0.001, 0.001) ;<br>*tau.v* ~ dgamma(0.001, 0.001);

The convolution model (Model III) containing the covariates and both the unstructured and spatially structured random effects was constructed as follows:

$Y_i \sim$ Poisson (μ_i);

where $Y_{i,j}$, the observed number of TB cases in county i, was assumed to follow a Poisson distribution with mean μ_i; and the log of the mean was modelled as:

$$Log\ (\mu i) = log\ (E_i) + \alpha + \beta k\ X_i k + \gamma\, t + U_i + V_i;$$

where E_i is the expected number of TB cases in area i, α is the intercept, β_k is the coefficient for covariate X_k, γ temporal trend in the outcome variable, t the quarters of the annual period (2013 2018), U_i are the unstructured random effects and V_i are the spatially structured random effects. The spatially structured random effects (V_i) were computed using a CAR structure by including an adjacency matrix to determine the spatial relationships between each pair of counties. The adjacency matrix for each county was generated using ArcGIS. A weight of 1 was given if two counties were neighbouring and a weight of 0 was given if two counties were not neighbouring. Two counties were considered to be neighbouring if they shared the same edges or corners (i.e. queen contiguity). Prior probability distributions for the coefficients (β) were assumed to have normal distributions with a mean =0 and a precision (i.e. inverse of variance) = 1 x 10^{-6}. For the intercept (α) a flat prior distribution was used (i.e. a non-informative, improper prior with bounds

- ∞ and +∞). The prior for the precision of the unstructured and spatially structured random effects was assigned a non-informative gamma distribution with shape and scale parameters = 0.001.
